# Reactive community-based self-administered treatment against residual malaria transmission: study protocol for a randomized controlled trial

**DOI:** 10.1186/s13063-018-2506-x

**Published:** 2018-02-20

**Authors:** Joseph Okebe, Joan Muela Ribera, Julie Balen, Fatou Jaiteh, Yoriko Masunaga, Davis Nwakanma, John Bradley, Shunmay Yeung, Koen Peeters Grietens, Umberto D’Alessandro

**Affiliations:** 1 Disease Control & Elimination Theme, Medical Research Council Unit, Fajara, The Gambia; 2 Medial Anthropology Research Center (MARC), Universitat Rovira i Virgili, Tarragona, Spain; 3 School of Health and Related Research, The University of Sheffield, Sheffield, UK; 4 Medical Anthropology Unit, Department of Public health, Institute of Tropical Medicine, Antwerp, Belgium; 5 Amsterdam Institute of Social Science Research, University of Amsterdam, Amsterdam, The Netherlands; 6 MRC Tropical Epidemiology Group, London School of Hygiene and Tropical Medicine, London, UK; 7 Faculty of Infectious and Tropical Diseases, London School of Hygiene and Tropical Medicine, London, UK; 8 Department of Disease Control, Faculty of Infectious and Tropical Diseases, London School of Hygiene and Tropical Medicine, London, UK

**Keywords:** Reactive case treatment, Community, *Plasmodium falciparum*, Formative research, Prevalence

## Abstract

**Background:**

Systematic treatment of all individuals living in the same compound of a clinical malaria case may clear asymptomatic infections and possibly reduce malaria transmission, where this is focal. High and sustained coverage is extremely important and requires active community engagement. This study explores a community-based approach to treating malaria case contacts.

**Methods/design:**

This is a cluster-randomized trial to determine whether, in low-transmission areas, treating individuals living in the same compound of a clinical malaria case with dihydroartemisinin-piperaquine can reduce parasite carriage and thus residual malaria transmission. Treatment will be administered through the local health system with the approach of encouraging community participation designed and monitored through formative research. The trial goal is to show that this approach can reduce in intervention villages the prevalence of *Plasmodium falciparum* infection toward the end of the malaria transmission season.

**Discussion:**

Adherence and cooperation of the local communities are critical for the success of mass treatment campaigns aimed at reducing malaria transmission. By exploring community perceptions of the changing trends in malaria burden, existing health systems, and reaction to self-administered treatment, this study will develop and adapt a model for community engagement toward malaria elimination that is cost-effective and fits within the existing health system.

**Trial registration:**

Clinical trials.gov, NCT02878200. Registered on 25 August 2016.

**Electronic supplementary material:**

The online version of this article (10.1186/s13063-018-2506-x) contains supplementary material, which is available to authorized users.

## Background

There is growing interest in mass drug administration (MDA) of at-risk populations with an effective antimalarial as a means to reduce the human reservoir of infection [1]. MDA aims to provide full treatment courses to the whole population to clear asymptomatic infections and provide posttreatment prophylaxis to prevent reinfection. It has been used effectively in the control and elimination of diseases such as trachoma and in emergencies such as malaria epidemics. The use of MDA is recommended in areas approaching interruption of transmission; with good access to treatment, effective vector control, and surveillance systems; and having a minimal risk of reintroduction of infection [2]. MDAs have been conducted using a variety of drug regimens at different dosages, timings, and frequencies. These show substantial but short-lived reduction in *Plasmodium falciparum* parasite carriage [3]. With the growing awareness of heterogeneity and clustering in transmission, MDA approaches have been modified by systematic (mass screening and treatment) or focused (focal screening and treatment) screening and treatment of populations. Reactive case detection (i.e., screening treating positive contacts in response to a clinical event) has been tested [4, 5] and implemented in some countries [6, 7]. However, its impact has been variable [8, 9] because it is affected by the sensitivity of the diagnostic tool and the radius of intervention around a clinical case [10, 11]. These programs require strong coordination and often lack community engagement.

Malaria transmission in The Gambia is seasonal, occurring during and immediately after the rainy season (July–December), and has decreased significantly over the past decade [12, 13]. Key interventions that have led to this decline are case management with artemisinin-based combination therapy (ACT) and the use of long-lasting insecticidal nets (LLINs) [14]. Malaria case management involves screening suspected cases, mostly by a rapid diagnostic test (RDT), and treatment of positive cases with artemether-lumefantrine. This service is available at all public health facilities and from village health workers (VHWs) at the community level. VHWs are volunteers identified by their communities, trained by the government, and provided with RDTs and artemether-lumefantrine.

As malaria becomes uncommon, community engagement is needed to help identify cases or treat asymptomatic infected individuals, and these steps depend on a robust understanding of community perceptions of disease, infection, and transmission [15, 16]. The planned study develops and implements a strategy for community engagement to reduce malaria prevalence and thus malaria transmission. Such an approach of reactive treatment of clinical malaria cases’ contacts is developed using a mixed methods approach to understanding how communities perceive it and how it can be integrated within the existing health system.

### Trial objective

The objective of this trial is to determine whether administering an antimalarial treatment to all individuals living in the same compound of clinical malaria cases would result in a lower prevalence of *P. falciparum* infection at the village level.

## Methods/design

**Fig. 1:**
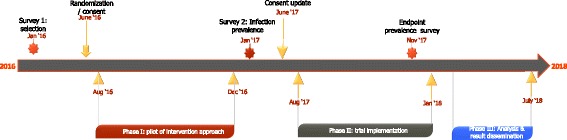
Timeline for project activities

This is a cluster-randomized trial designed to assess the impact of reactive treatment of contacts of a clinical malaria case living in the same compound on the prevalence of malaria infection at the end of the transmission season. The trial is planned over two transmission seasons. In the first (exploratory) season, villages are identified and approaches to integrate the intervention into the community and health systems will be tested and adapted by social scientists and health systems researchers. The final portfolio of messages to support the intervention will be applied in the second (implementation) season (Fig. 1).

### Study setting

The study is being conducted in villages on the North Bank of the River Gambia and covers an area of approximately 45 km west of the town of Farafenni. *P. falciparum* is responsible for nearly all malaria infections in The Gambia [17]. Malaria transmission in this area has declined substantially, with parasite prevalence of about 3% in a population sampled during the peak of transmission [13]. It is therefore an ideal location because the intervention is designed for targeting residual clusters of transmission.

A village is composed of compounds, and each compound is clearly defined by a fence. It is typically composed of several buildings and rooms with different functions that serve one or several households. Locally, a household is defined as “all the people who eat from the same bowl” and consists of members of an extended patrilineal family. A typical compound has the men’s house, where each adult male has his own room; the women’s house, where the wives of all married men from the household, their daughters, and children stay; the kitchen; and the “boys’ house” for young circumcised men. The decision to treat all individuals in a compound was based on the need to cover as many individuals as possible around the clinical case and also because of the clear demarcation between compounds.

### Village selection and consenting

**Fig. 2:**
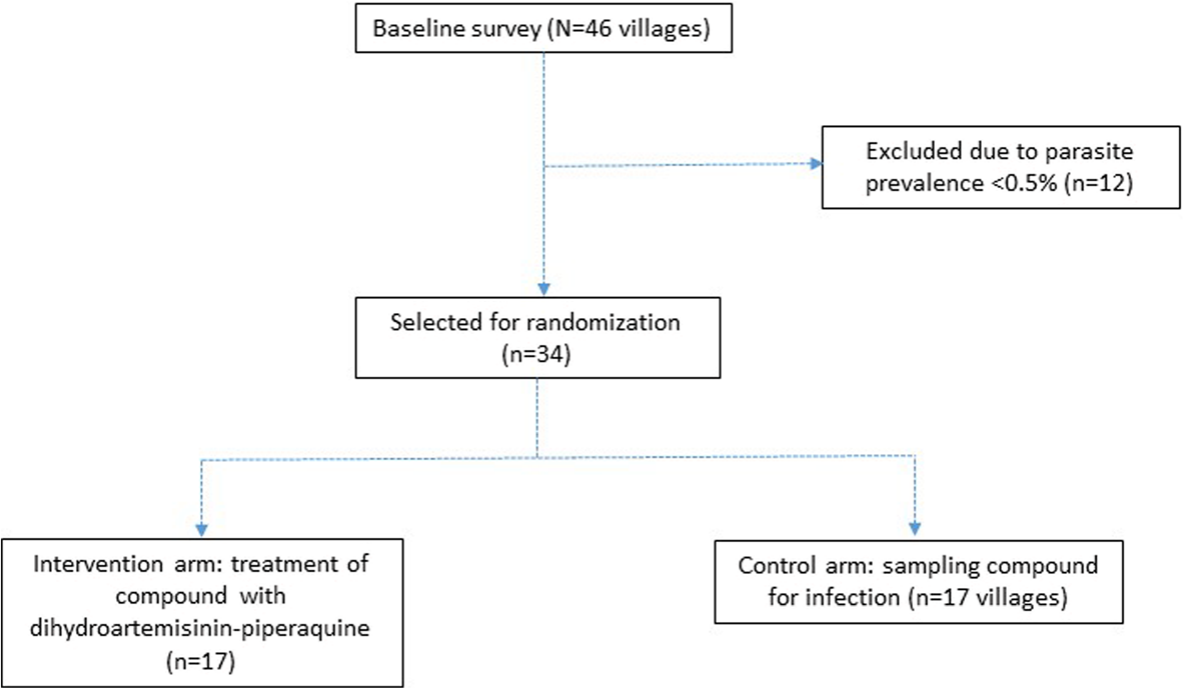
Village selection and randomization into trial arms

Medium-sized villages (population ≥ 100) in the area were targeted, and parasite prevalence by loop-mediated isothermal amplification (LAMP) assay was determined [18] in January 2016. Villages of this size were targeted because they are likely to have a resident VHW. Villages with a parasite prevalence > 0.5% were included in the trial and randomly assigned to an intervention or control arm using a computer-generated minimizing algorithm (Fig. 2). A census was conducted in each village to determine the population size and age structure. In addition, weight measurements were documented for residents in villages that made up the intervention arm.

Because all residents of the villages are potentially at risk of infection and clinical malaria during the transmission season, a written informed consent will be required from everyone before study-related activities are implemented. To ease identification of compound members, consent will be obtained preemptively before the start of the malaria transmission season in all selected villages. Consent for children will be provided by their guardians, and those 12–17 years old will be asked to provide an assent in addition to a written consent from a responsible adult. This process will be updated in the course of the trial, especially in compounds where new residents have been added or individuals missed in the earlier exercise are reported. A list of residents in the study villages, by compound, including weight and consent status, will be generated and made available to study nurses at the local health facility and to VHWs involved in the trial.

### Activities in the intervention arm

Trial activities are initiated after an individual from a village assigned to intervention is diagnosed with malaria and treated at either a health facility or by a VHW with artemether-lumefantrine. Individuals living in the same compound as this index case will be identified from the census logbook produced for the trial, with the help of the patient or escort if the patient is a minor. A full (3-day) course of dihydroartemisinin-piperaquine (DP), dispensed on the basis of body weight, will be packed for each individual in the compound for distribution. DP is recommended for the treatment of uncomplicated *P. falciparum* malaria [19], available in pediatric (dihydroartemisinin 20 mg/piperaquine 160 mg) and older child/adult (dihydroartemisinin 40 mg/piperaquine 320 mg) tablet formulations, and administered as a once-daily treatment.

**Fig. 3:**
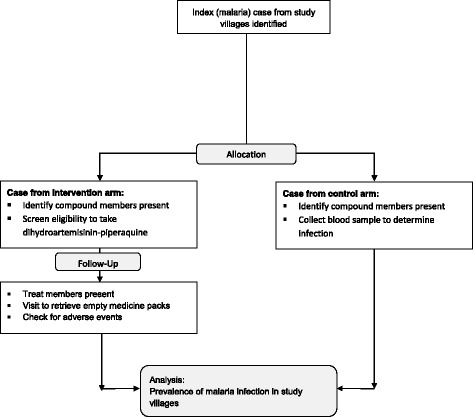
Schematic describing activities in the trial arms following identification of a malaria case

Where the clinical case is identified at a health facility, study nurses will hand treatment packs labeled with each recipient’s name and dosing instructions to the escort to take home. The study nurse informs the resident VHW or village collaborator (VC)—a volunteer identified by the community, if the village does not have a VHW—of the event and the compound where treatment has been sent. The VHW or VC then visits the compound on the same day and helps distribute the medicines. If the malaria case is detected by the VHW, he/she will visit the compound and distribute prepacked medicines to residents of the compound following instructions on the logbook developed in a way usable for VHWs. Follow-up visits will be scheduled for the day after treatment has been completed where the VHW or VC returns to the compound to confirm that treatment was taken, retrieve empty or unused drug packets, and inquire about any adverse events during the period. This information will be relayed back to the study nurse. A field assistant will collect any unused drugs and empty packets (Fig. 3).

**Fig. 4:**
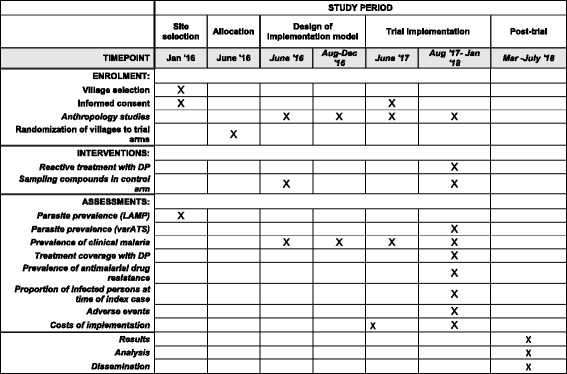
Table showing schedule for enrollment, interventions, assessments, analysis, and dissemination of results

### Activities in the control arm

Activities in the control arm during the trial implementation period will be limited to observations of the routine case management of malaria by either the VHW or the health facility and measurement of the risk of infection in the compound of an index case. Where an index case is confirmed, it is reported to the study team that will visit the compound and collect a fingerprick blood sample for later detection of *P. falciparum* infection at the laboratory. Members of the compound are not treated.

### Eligibility and participation

The unique requirement for participation in the trial informs the need for an adaptive approach in the study design and defining eligibility. All residents in the intervention villages are potentially eligible. However, reactive treatment targets only compounds in the intervention villages where a clinical malaria case occurs and treats only compound members who are eligible at that time. The key eligibility criteria for receiving treatment include residence in the study area for at least 1 month during the transmission season, willingness to participate, weight ≥ 5 kg, and no known history of heart disease or allergies to DP; pregnant women are eligible only after the first trimester. Eligibility of compound members may change during the course of the trial period, so it is essential to ensure comprehension and maintain interest in the trial. For instance, pregnancy (first trimester) and weight (≥ 5 kg) status need to be confirmed at the time of treatment. Social scientists involved with the trial will explore perceptions of treatment for asymptomatic malaria infections and produce information that provides clarity and context to the trial activities, which will be shared with the communities during the implementation phase.

In the control arm, only a blood sample from the compound members will be collected. Therefore, the eligibility criteria are limited to residence in the study area for at least 1 month during the transmission season and willingness to participate.

### Study outcomes

The primary endpoint is the prevalence of *P. falciparum* malaria infection measured by molecular methods at the end of the second year. Prevalence will be compared between intervention and control arms.

Secondary endpoints include the following:

- Prevalence of clinical (laboratory-confirmed) malaria detected in study villages during the transmission season
- Treatment coverage: the proportion of individuals in each compound in the intervention arm that received and took the prescribed dose
- Prevalence of antimalarial drug resistance molecular markers and difference between study arms
- In the control arm, the proportion of infected individuals at the time of a clinical event

### Sample size calculation

The sample size calculation assumed reactive treatment would result in a 60% difference in parasite prevalence between the intervention and control arms in an area with a prevalence around 5% [20]. A minimum of 16 villages per trial arm would be needed to detect such a reduction between the intervention and control arms with 80% power, a 5% significance level, and a coefficient of variation of 0.7 [21].

### Assignment of villages to trial arms

The unit of randomization is the village because the primary endpoint, malaria prevalence, is measured at this level. Using the data from a baseline survey in which blood samples were analyzed with a LAMP assay, the trial statistician will randomize villages to one of the two study arms using a computer-generated randomization sequence. First, a minimization algorithm [22] based on the imbalance measure by Raab and Butcher [23] will be used to generate a series of optimal scenarios that adjust for imbalances in prevalence between arms. Villages will then be randomly allocated, using a two-block randomization sequence, into two groups by selecting an optimal sequence from the lot generated. This will be done using Stata 14 software (StataCorp, College Station, TX, USA). Blinding of interventions is not feasible, because both individuals taking the medicines and health workers distributing them will be aware of who is receiving the intervention. However, samples collected during the study will be assigned unique identifiers, and staff processing samples will not be aware of the identity of the individuals or their allocation arm.

### Laboratory processes

Fingerprick blood samples collected on filter papers during the prevalence surveys and compound screening will be tested for *P. falciparum* infection by using a qPCR assay targeting the *var* gene acidic terminal sequence (varATS) [24]. This assay is highly sensitive and hence useful for the detection of low-density malaria infections that are usually found in low-transmission settings. Positive samples will be screened for molecular markers of drug resistance, namely the *Pfcrt*-76 and *Pfmdr1*-86 gene polymorphisms, using the established high-resolution melting protocols [25].

### Data collection, management, and analysis

Data from the census of the villages and measured weights will serve as the platform for trial activities and will be stored in a retrievable format in a Microsoft Access database (Microsoft Corp., Redmond, WA, USA) set up on electronic tablets. Information on the confirmed case will be used to identify the compound of the index case and generate a list of persons for treatment and follow-up. In the control villages, forms will be generated that are based on the compound of the malaria case for persons from whom to collect a blood sample for parasite detection. Completed forms will be double-entered into an OpenClinica database (OpenClinica LLC, Waltham MA, USA). Results derived from the analysis of samples from the prevalence surveys will be linked to the main trial database for the analysis. All staff involved in the trial will be trained on their specific tasks.

The primary endpoint, *P. falciparum* prevalence, defined as the proportion of sampled residents carrying parasites detectable by varATS PCR, will be compared between study arms using generalized estimating equations to account for within-cluster correlations. Random-effects multivariable logistic regression models will be used to analyze binary outcomes, and mixed linear regression will be used for normally distributed continuous outcomes. An interim analysis of results is not planned, and the analysis plan will be produced and agreed on before the close of the trial database.

For the cost-effectiveness analysis, we will describe the costing of the trial and estimate the costs of implementing similar interventions at a programmatic scale. This takes into account the existing infrastructure and a projection of any additional resources required to be able to deliver the interventions outside a trial setting and integrate them into the existing health system, including the National Malaria Control Program and community-based health services. We will run a series of sensitivity analyses, taking into account various possible inputs and processes for the interventions’ scaling-up (Fig. 4).

### Quality management

A data and safety monitoring board (DSMB), independent of the sponsor, will be appointed to advise the sponsor and investigators on safety issues in the trial. During the trial, study nurses and VHWs will be trained to check and report all events associated with treatment with DP. All adverse or serious adverse events will be reported to the local ethics committee and the DSMB, based on reporting schedules stated in the protocol (Additional file 1).

The study will apply a transdisciplinary approach in the conduct of the trial with a team comprising epidemiologists, social scientists, health systems researchers, and health economists. A prototype for intervention, designed by the trial team, will be tested and adapted in the first year using feedback from researchers, relevant community stakeholders, health service providers, and policy makers according to their knowledge and experience. Perceptions, understanding, and messages during the implementation stage will be monitored systematically with implementation problems dialogically tackled by the trial team.

The trial is implemented through the Gambian health system, including VHWs supervised by community health nurses on routine tasks and by study staff on trial-specific procedures. Study nurses will be based at selected health facilities and will rely on the active participation of identified key stakeholders in each intervention community, patients with malaria, and their compound members for implementation and feedback.

### Confidentiality of trial data and result dissemination

Information on study participants will remain confidential. Unique identifiers will be used on case report forms (CRFs) and filter papers; CRFs will be kept in locked files; and electronic data on tablets will have password securities accessible only to authorized study team members. All identifiable information will be delinked from data before being transferred to the study database. The results of the trial will be shared with The Gambia’s Ministry of Health and Social Welfare through the National Malaria Control Program and with the participating communities.

## Discussion

Malaria morbidity and mortality have declined below levels previously thought unachievable in sub-Saharan Africa [14]. This was made possible by the increased access to effective interventions such as LLINs and ACTs. Despite this, the risk of malaria transmission persists owing to a large human reservoir of infection that consists mainly of asymptomatic infections [26]. Therefore, reducing the prevalence of infections is required to sustain the current gains and eventual elimination of the disease.

Countries that have eliminated malaria have been successful mainly by controlling the mosquito vector and by MDA [27, 28]. Although there is renewed interest in use of MDAs, contemporary reviews on their utility for malaria elimination raises questions regarding their cost-effectiveness, sustainability, the increased drug pressure, and the potential for the emergence of drug-resistant parasites [3, 27]. This has led to proposing focused approaches such as mass screening and treatment, although available field diagnostic tests are not sufficiently sensitive to detect low-density infections [11]. These programs require a huge commitment and effort by the health system, and high coverage of these interventions is difficult to sustain without engagement of the local community.

Community engagement and participation has played a critical role in malaria control [29, 30] and elimination [31]. However, it is challenging to maintain enthusiasm and participation when the perception of the risk of disease is declining. Studies to identify and evaluate ways to engage communities to ensure sustained and high coverage of community-targeted interventions are needed. The use of an iterative investigatory approach into the evolving local sociocultural, behavioral, and practical issues that affect malaria prevention and treatment has been proposed [16], and this could be applied in the context of a malaria elimination program targeting asymptomatic infections [32, 33].

In this trial, we build on the already established concept of passive case detection to target for treatment individuals around the index case, and this will be done with the active involvement of patients, their family members, and existing community organizations to optimize adherence. The treatment for compound members will be delivered either by the patients themselves or by the local VHW. Distribution within the compound will be implemented by the VHW or VC trained by the study team, and the process will be overseen by the head of the compound. The study team, via the VHW or VC, will monitor treatment compliance. In The Gambia, the compound is a recognizable spatial unit and a cluster of persons with similar exposure to mosquito bites and treatment-seeking behavior [34]. It is assumed that the systematic treatment of both cases and compound contacts should reduce or eliminate the reservoir of infection and hence decrease or interrupt malaria transmission. Formative research will provide precious information on the best approach to optimizing the intervention by focusing on “what” (messages, form, and content), “how” (tools and strategies), “when and where”, and “who” (institutions and individuals). The study is designed with the goal of developing a model that can be rapidly operationalized by national malaria control programs.

## Trial status

The current protocol is version 3.0 (15 February 2017). Field activities for the exploratory phase were conducted between August and December 2016. The implementation stage ran from August 2017 to January 2018. The end of the study survey was planned in December 2017. Trial results are expected in July 2018. Trial is ongoing.

## Additional file

Additional file 1: Standard Protocol Items: Recommendations for Interventional Trials (SPIRIT) 2013 checklist: recommended items to address in a clinical trial protocol and related documents. (DOC 59 kb)

## Abbreviations

ACT: Artemisinin-based combination therapy
CRF: Case report form
DP: Dihydroartemisinin-piperaquine
DSMB: Data and safety monitoring board
LAMP: Loop-mediated isothermal amplification
LLIN: Long-lasting insecticidal net
MDA: Mass drug administration
RDT: Rapid diagnostic test
varATS: *var* Gene acidic terminal sequence
VC: Village collaborator
VHW: Village health worker
